# Clinicians’ perceptions and practices of diagnostic assessment in psychiatric services

**DOI:** 10.1186/s12888-023-04689-w

**Published:** 2023-03-23

**Authors:** Benjamin Bohman

**Affiliations:** 1 Centre for Psychiatry Research, Department of Clinical Neuroscience, Karolinska Institutet & Stockholm Health Care Services, Region Stockholm, Stockholm, Sweden; 2 Centre for Psychiatry Research, Liljeholmstorget 7, Stockholm, SE- 117 63 Sweden

**Keywords:** Diagnostic techniques and procedures, Health care quality assessment, Quality improvement

## Abstract

**Background:**

Diagnostic assessment in psychiatric services typically involves applying clinical judgment to information collected from patients using multiple sources, including anamnesis and structured diagnostic interviews. Research shows that clinicians’ perceptions of diagnostic assessment are associated with their diagnostic practices, and that perceptions and practices may vary according to clinician characteristics. Examining clinicians’ perceptions and practices of diagnostic assessment is important for quality improvement in psychiatric services, including implementation of evidence-based practice procedures. The purpose of the present study was to evaluate clinicians’ perceptions and practices of diagnostic assessment in psychiatric services and examine whether these perceptions and practices varied according to profession and age, with the aim of providing a basis for quality improvement.

**Methods:**

A total of 183 (53.2%) clinicians in community-based adult psychiatric services in Stockholm, Sweden participated in an online survey. Differences between professions were analyzed using Kruskal-Wallis tests and effect sizes were calculated. Associations of clinicians’ perceptions with their age were examined using Spearman correlations.

**Results:**

Overall, clinicians had positive attitudes toward diagnostic assessment, and they considered themselves as competent. Differences were as most pronounced between nurses and other professions. Nursed had conducted fewer assessments, perceived themselves as less competent, and reported to a smaller extent to be able to determine which diagnosis should be the target for treatment in patients with multiple diagnoses. There were no associations of clinicians’ perceptions with their age. Some potential areas of improvement were identified, including clinician qualifications, education in diagnostic assessment, and contents of diagnostic assessment.

**Conclusions:**

The results of the present study may provide a basis for quality improvement in psychiatric services. For example, it may be important to pay attention to potential differences in perceptions and practices between professions in efforts to improve quality of assessment and care.

## Introduction

In psychiatric services, assigning a diagnosis is often required by clinics and third-party payers to authorize treatment, and diagnoses aid in treatment planning as many treatments are designed for specific psychiatric disorders [1]. Typically, diagnostic assessment involves applying clinical judgment to information collected from patients using multiple sources, including anamnesis, structured diagnostic interviews, self-report measures, and neuropsychological testing [2]. For example, to assign a diagnosis the Mini International Neuropsychiatric Interview (MINI; [3]) and the Structured Clinical Interview for Diagnostic and Statistical Manual of Mental Disorders (SCID; [4]) are widely used internationally [5, 6], and the Patient Health Questionnaire-9 (PHQ-9; [7]) is a commonly recommended measure to assess depressive symptoms [8]. Together with evidence-based treatment, evidence-based assessment, including diagnostic assessment, is considered an integral part of evidence-based practice [9, 10].

Studies show that clinicians’ perceptions of diagnostic assessment are associated with their diagnostic practices. For example, positive associations have been found of clinician attitudes toward standardized assessment (SA; e.g., structured diagnostic interviews, outcome measures) and the utility of diagnosis with their self-reported practices, including frequency of diagnostic assessment and use of SA measures [1, 11]. Furthermore, these and other studies [12, 13] show that clinicians’ perceptions or practices of diagnostic assessment may vary according to clinician characteristics, such as profession, theoretical orientation, and practice setting. In addition to clinicians’ perceptions of diagnostic assessment being associated with their diagnostic practices, providing training in the use of SA may improve clinicians’ positive attitudes, skill, and use of SA measures [14]. In turn, clinicians’ diagnostic practices provide information about the extent to which clinicians follow evidence-based clinical guidelines of assessment, with studies showing that SA is used infrequently in psychiatric services [11, 15, 16]. For example, a study found that clinicians used structured diagnostic interviews with only 14.8% of their patients, and that 51.6% reported never using diagnostic interviews with any of their patients [12]. Thus, examining clinicians’ perceptions and practices are important for quality improvement in psychiatric services, including implementation of evidence-based practice procedures.

In community-based adult psychiatric services in Stockholm, Sweden, diagnostic assessment as presently conducted was introduced in 2012. However, it has never been evaluated, so it is not known what clinicians think of diagnostic assessment and how they practice it. The purpose of the present study was to evaluate clinicians’ perceptions and practices of diagnostic assessment in this setting with the aim of providing a basis for quality improvement. Research questions were: (1) What attitudes do clinicians have toward diagnostic assessment?, (2) What beliefs do they hold of its purpose, contents, and procedure?, (3) How do they proceed in conducting diagnostic assessment?, (4) To what extent do clinicians consider themselves as competent in diagnostic assessment and do they perceive a need of education?, and (5) Are there differences in perceptions or practices according to profession and age?

## Methods

### Design and setting

The present study was designed for clinical evaluation of the implementation of a specific diagnostic assessment procedure. The study was commissioned by the management of the community-based psychiatric services in Stockholm and conducted cross-sectionally using an online survey. A steering group, headed by the author, was created to conduct the evaluation. The group consisted of one representative from each of six participating clinics and two individuals from the Centre for Psychiatry Research in Stockholm. The representatives were appointed by the heads of the clinics. Of the eight group members, two were physicians specialized in psychiatry (i.e., psychiatrists), five clinical psychologists, and one was a psychiatric nurse. In addition, five of the members were section managers, and five had a PhD degree.

### Sample selection of services

In Stockholm, community-based adult psychiatric services are organized into eight clinics, which provide both inpatient and outpatient care. Five of the clinics provide services for a range of psychiatric disorders and populations and are geographically spread out across the Stockholm region, whereas the other three clinics exclusively provide services to patients with substance use disorders, patients with eating disorders, and forensic psychiatric patients, respectively. In the present study, clinicians from six clinics participated: four of the clinics geographically spread out across the Stockholm region, and the clinics for substance use disorders and eating disorders. The clinic located in the southwest of Stockholm was not eligible for participation because it recently had started using a different diagnostic assessment procedure than the rest of the clinics, and the forensic psychiatry clinic was not invited to participate due to the involuntary nature of its activities.

### Diagnostic assessment in psychiatric services in Stockholm

In 2012, a “basic” diagnostic assessment using a set battery of SA measures was introduced for all individuals who are referred to community-based adult psychiatric services in Stockholm. The basic assessment is mandatory and the purpose is to provide a basis for assigning a diagnosis and planning treatment according to local clinical guidelines [17]. The basic assessment includes collecting anamnestic information and conducting a structured diagnostic interview using the MINI or SCID. In addition, patients complete a number of self-report measures: the PHQ-9 for depressive symptoms, Adult ADHD Self-Report Scale (ASRS; [18]) for symptoms of attention-deficit/hyperactivity disorder (ADHD), Alcohol Use Disorders Identification Test-Consumption (AUDIT-C), which consists of three items about alcohol consumption from the AUDIT [19], and the EuroQol Five Dimensional Questionnaire (EQ-5D; [20]) for health-related quality of life. Finally, the assessment includes the Clinical Global Impression-Severity (CGI-S; [21]), which is a clinician-administered measure consisting of a single item assessing the severity of a patient’s illness. The clinics decide themselves which clinicians conduct the basic assessment. Typically, the basic assessment is conducted by one clinician, and upon its completion an appointment is made with another clinician for initiation of pharmacological and/or psychological treatment. However, sometimes several clinicians are involved in conducting different parts of the assessment. The results of the basic assessment (i.e., anamnestic and diagnostic information, and scores on self-report measures) are documented in patients’ medical records. Prior to initiating treatment, clinicians consult the medical records for information on the basic assessment. In the pre-pandemic years of 2018 and 2019, an average of 9350 basic assessments were conducted per year across participating clinics [22].

If the basic assessment is considered insufficient to assign a diagnosis, or it can be suspected that patients are suffering from intellectual disability or neurodevelopmental disorders (autism spectrum disorder or ADHD), the assessment is extended to include additional structured diagnostic interviews and other measures, including neuropsychological testing. There are no guidelines specifying the purpose of the “extended” assessment; in the present study the purpose was described as providing a more elaborate basis for assigning a diagnosis and planning treatment. The extended assessment is often conducted by a clinician with expertise in the suspected disorder and its assessment. In contrast to the basic assessment, the extended assessment is not standardized and can include whatever measures clinicians find relevant.

### Participants

Eligible participants were clinicians of any profession who had documented in the medical record system that they had conducted at least one basic assessment during the last 12 months. (The extended assessment is not documented in a similar systematic manner.) A total of 183 participants responded to the survey of the 344 clinicians who were eligible for participation, representing a response rate of 53.2%. Mean age was 46.0 years, and most participants (62.3%) were female. The three most common professions were physicians (48.1%), clinical psychologists (30.6%), and nurses (10.9%). For characteristics of participants, see Table 1.

**Table 1:** Characteristics of Participants

		*n* (%)
Participants		183 (53.2)
Age, *M* (*SD*), range		46.0 (10.9), 28–76
Gender		
	Female	114 (62.3)
	Male	66 (36.1)
	Other	1 (0.5)
	Prefer not to say	2 (1.1)
Profession		
	Physician specialized in psychiatry	51 (27.9)
	Resident physician specializing in psychiatry	37 (20.2)
	Clinical psychologist	56 (30.6)
	Psychiatric nurse	16 (8.7)
	Nurse, other	4 (2.2)
	Psychiatric aide	7 (3.8)
	Medical social worker	2 (1.1)
	Occupational therapist	1 (0.5)
	Other profession	9 (4.9)
Work tasks		
	Clinical work^a^	167 (91.3)
	Responsibility for a process of care^b^	23 (12.6)
	Specialist physician with medical managerial responsibility	14 (7.7)
	Manager	3 (1.6)
	Other work tasks	14 (7.7)
Clinic		
	Clinic for substance use disorders	52 (28.4)
	Clinic in the south of Stockholm	44 (24.0)
	Clinic in the north of Stockholm	42 (23.0)
	Clinic in the northwest of Stockholm	28 (15.3)
	Clinic in the south of Stockholm (Södertälje)	12 (6.6)
	Clinic for eating disorders	5 (2.7)
Process of care^c^		
	Attention-deficit/hyperactivity disorder	23 (17.8)
	Emotionally unstable personality disorder	22 (17.1)
	Posttraumatic stress disorder	19 (14.7)
	Bipolar disorder	13 (10.1)
	Anxiety disorders	10 (7.8)
	Psychotic disorders	9 (7.0)
	Obsessive-compulsive disorder and body dysmorphic disorder	6 (4.7)
	Autism spectrum disorder	5 (3.9)
	Eating disorders	4 (3.1)
	Addictive disorders	4 (3.1)
	Depressive disorders	3 (2.3)
	Other process of care	11 (6.0)

^a^ Diagnostic assessment, or pharmacological or psychological treatment.

^b^ A process of care refers to an area of clinical focus and expertise. Typically, clinicians conducted assessment within a single process of care.

^c^ The process of care within which participants (*n* = 129) who have conducted the extended assessment during the last 12 months most often conducted the assessment.

In previous similar studies in which no “sensitive” personal data (e.g., health status, ethnicity) were collected the Swedish Ethical Review Authority has judged that the studies were not subjected to the Swedish Ethical Review Act; thus, no ethical approval was sought for the present study. However, the study was carried out in accordance with the World Medical Association Declaration of Helsinki, and national guidelines and regulations. Specifically, risk assessment was conducted, participant privacy was protected, and data were stored on secure servers. Participants provided informed consent by reading study information and clicking on the link to the online survey. Participation was voluntary and anonymous. All heads of participating clinics approved of the study.

### Assessment

The purpose of the survey was to provide specific information on clinicians’ perceptions and practices of the basic and extended diagnostic assessments within psychiatric services in Stockholm. Thus, the steering group developed the survey questions based on their knowledge and experience of the assessments, and feedback they had received from other clinicians conducting diagnostic assessment in this setting. The survey consisted of 39 questions and was administered in an online format using Webropol (www.webropol.com). Questions covered the following areas: characteristics of participants, characteristics of the assessments (e.g., number of appointments), purpose of diagnostic assessment, contents of the basic assessment, perceived competence and need of education in diagnostic assessment, and perceived importance of consulting other clinicians’ assessments prior to starting treatment of patients. Responses were made on a Likert-type scale from 1 (*Not at all*) to 5 (*To a very large extent*), with an intermediate anchor at 3 (*To a moderate extent*).

### Procedure and analysis

An individual independent of the study working with information systems within psychiatric services in Stockholm accessed contact information to eligible clinicians and distributed study information and a link to the survey using an email program. Participants were informed that the individuals analyzing the data would not have access to information on who provided specific responses (e.g., Internet protocol address), and that participation was voluntary and anonymous. Participants were also informed that their responses were to be compiled in a report to which they would have access. Emails were sent in March 2022 to eligible clinicians at five of the clinics, who could participate during this period. Emails were sent to clinicians at the sixth clinic in May 2022, inviting them to participate during this period. Three reminders were sent, the first reminder five days following the initial study invitation, then reminders were sent every third day. The survey took 15–20 min to complete.

Differences between physicians, psychologists, and nurses were examined using Kruskal-Wallis tests. These professions represented 89.6% of the sample; other professions were too diverse in terms of education (e.g., psychiatric aides, psychotherapists) to be combined into a single category and were thus not included in analysis of group differences. Pairwise comparisons with adjusted *p*-values were conducted, with statistical significance set at *p* ≤ .050. Effect sizes for pairwise comparisons were calculated as *r* = *z* / √*N*, in which *z* is the standardized test statistic and *N* is the total sample size on which *z* is based. *r*-values of 0.10, 0.30, and 0.50 were interpreted as small, medium, and large, respectively. Associations of clinicians’ perceptions with their age were investigated using Spearman’s correlation coefficient *r*_*S*_. Data were analyzed using the SPSS (Version 28, SPSS Inc., Chicago, IL).

## Results

### The basic diagnostic assessment

A total of 78.7% (*n* = 144) of the participants believed to a large (a score of 4) or very large (a score of 5) extent that the basic assessment fulfilled its purpose of providing a basis for assigning a diagnosis and planning treatment (for means of the basic assessment, see Table 2). There were no significant differences between professions (for comparisons between professions, see Table 3). The mean number of appointments for conducting the basic diagnostic assessment was 2.9 (*SD* = 1.4) appointments. On average, participants had been involved in 12.3 basic assessments during the last 12 months; however, the number of assessments varied greatly across participants and professions (Table 2). There were significant differences in the number of assessments involved in between physicians, psychologists, and nurses; pairwise comparisons showed a significant difference of a small to medium size between physicians and psychologists, indicating that physicians had been involved in more basic assessments than psychologists (Table 3). On average, participants had conducted 9.7 basic assessments on their own during the last 12 months; again, the number of assessments varied greatly across participants and professions (Table 2). There were significant differences in the number of assessments conducted on their own between professions; pairwise comparisons showed a significant difference of small to medium size between physicians and nurses, indicating that physicians had conducted more basic assessments on their own than nurses (Table 3).

**Table 2:** Responses to Statements About the Basic and Extended Diagnostic Assessments

	Basic				Extended			
	*M* (*SD*)				*M* (*SD*)			
	Total	Physicians	Psychologists	Nurses	Total	Physicians	Psychologists	Nurses
Assessment fulfills its purpose^a^	4.2 (0.9)	4.1 (1.0)	4.4 (0.8)	4.1 (1.0)	4.6 (0.7)	4.6 (0.8)	4.6 (0.6)	4.9 (0.4)
Number of assessments involved in	12.3 (23.4)	16.9 (26.5)	7.7 (19.4)	9.7 (24.1)	9.5 (13.3)	11.5 (15.2)	9.6 (12.2)	2.3 (3.8)
Number of assessments conducted on their own	9.7 (15.2)	10.8 (14.6)	9.6 (17.7)	3.1 (5.7)	10.0 (14.9)	7.5 (14.6)	13.7 (15.7)	3.9 (5.0)
I am competent in conducting assessment^a^	4.5 (0.7)	4.6 (0.7)	4.7 (0.5)	4.0 (1.1)	4.3 (0.8)	4.1 (0.8)	4.5 (0.6)	3.8 (1.8)
I need education in assessment^a^	2.3 (1.3)	2.3 (1.3)	2.1 (1.2)	2.7 (1.4)	2.9 (1.3)	2.9 (1.3)	2.7 (1.3)	2.4 (1.5)
I can determine which diagnosis should be the target of treatment^ab^	4.3 (0.9)	4.5 (0.7)	4.3 (0.7)	3.4 (1.4)	4.4 (0.8)	4.5 (0.8)	4.5 (0.5)	3.8 (1.5)
Importance of consulting other clinicians’ assessments^a^	4.3 (0.9)	4.2 (0.9)	4.3 (0.8)	4.8 (0.4)	4.6 (0.7)	4.6 (0.7)	4.6 (0.7)	4.8 (0.5)

^a^ Responses were made on a Likert-type scale from 1 (*Not at all*) to 5 (*To a very large extent*).

^b^ For basic assessment: “If I have conducted the MINI or SCID and collected anamnestic information”; for extended assessment: “If I have conducted the assessment on my own or substantial parts of it together with others”.

**Table 3:** Kruskal-Wallis Tests and Pairwise Comparisons of Differences Between Professions of the Basic Assessment

	Kruskal-Wallis test			Physicians-psychologists		Physicians-nurses		Psychologists-nurses	
	*H*	*df*	*p*	*p*	*r*	*p*	*r*	*p*	*r*
Assessment fulfills its purpose	2.04	2	0.361						
Number of assessments involved in	9.85	2	0.007	0.017	0.23	0.088	0.21	1.00	0.03
Number of assessments conducted on their own	9.28	2	0.010	0.815	0.09	0.007	0.29	0.092	0.25
I am competent in conducting assessment	7.57	2	0.023	0.966	-0.08	0.081	0.22	0.018	0.32
I need education in assessment	2.87	2	0.238						
I can determine which diagnosis should be the target of treatment^a^	13.20	2	0.001	0.368	0.13	0.001	0.35	0.047	0.28
Importance of consulting other clinicians’ assessments	8.74	2	0.013	1.00	-0.08	0.009	-0.29	0.070	-0.27

^a^“If I have conducted the MINI or SCID and collected anamnestic information”.

Three quarters of the participants (74.9%, *n* = 137) lacked specific education in the basic assessment. Nonetheless, a majority (89.6%, *n* = 164) considered themselves as competent to a large or very large extent in the basic assessment, and, consequently, a minority (19.1%, *n* = 35) believed to the same extent that they needed education in the assessment (Table 2). There were significant differences in perceived competence between professions; pairwise comparisons showed a significant difference of a medium to large size between psychologists and nurses, indicating that psychologists reported higher competence than nurses (Table 3). However, there were no significant differences in the perceived need of education between professions.

If the participants themselves had conducted the MINI or SCID and collected anamnestic information and the results showed that patients met criteria for multiple diagnoses, 84.1% (*n* = 153) believed to a large or very large extent that they could determine which diagnosis should be the target of treatment (i.e., the principal diagnosis), see Table 2. There were significant differences in this perceived ability between professions; pairwise comparisons showed significant differences of a medium to large size between physicians and nurses, and of a small to medium size between psychologists and nurses, indicating that physicians and psychologists reported higher ability than nurses (Table 3).

Most participants (82.2%, *n* = 148) considered it important to a large or very large extent to consult other clinicians’ basic assessments prior to starting treatment of patients (Table 2). There were significant differences between professions; pairwise comparisons showed a significant difference of a small to medium size between physicians and nurses, indicating that nurses believed this to be more important than physicians (Table 3). Concerning the basic assessment there were no significant associations of clinicians’ age with their perceived competence, need of education, ability to determine the diagnosis for treatment, or importance of consulting other clinicians’ basic assessments, neither for the entire sample, *r*_*S*_s = 0.01 - -0.05, *p*s = 0.487-0.941, nor separately for physicians, psychologists, and nurses, *r*_*S*_s = -0.03-0.32, *p*s = 0.118-0.857.

Considering the purpose of the basic assessment, participants assigned varying relevance to the measures included (Table 4). Most participants believed that anamnestic information (96.2%) and the MINI (84.9%) were relevant to a large or very large extent, whereas a minority (41.9%) believed the same of the SCID (although 30.1% of participants could not or preferred not to respond about the SCID). The proportion of participants using the MINI (97.8%) was almost twice as large as the proportion using the SCID (50.8%). The AUDIT-C and PHQ-9 were considered as the most relevant self-report measures, whereas CGI-S and EQ-5D were considered as the least relevant measures.

**Table 4:** Relevance and Removal of Measures of the Basic Diagnostic Assessment

	Relevance of measures *M* (*SD*)^a^	Relevance of measures *n* (%)^b^	Removal of measures *n* (%)
Mini International Neuropsychiatric Interview	4.4 (0.9)	152 (84.9)	14 (23.3)
Structured Clinical Interview for Diagnostic and Statistical Manual of Mental Disorders	3.5 (1.4)	39 (41.9)^c^	15 (25.0)
Anamnestic information	4.9 (0.4)	176 (96.2)	0
Patient Health Questionnaire-9	3.9 (1.1)	127 (69.4)	16 (26.7)
Adult ADHD Self-Report Scale	3.9 (1.2)	125 (68.3)	18 (30.0)
Alcohol Use Disorders Identification Test-Consumption	4.5 (0.8)	154 (84.2)	3 (5.0)
EuroQol Five Dimensional Questionnaire	2.8 (1.2)	45 (24.6)	35 (58.3)
Clinical Global Impression-Severity	3.1 (1.3)	66 (36.1)	29 (48.3)

^a^ Responses were made on a Likert-type scale from 1 (*Not at all*) to 5 (*To a very large extent*).

^b^ Response alternatives 4 and 5 in combination.

^c^ Of the participants, 30.1% (*n* = 28) could not or preferred not to respond.

A third (32.8%, *n* = 60) of the participants believed that some measures of the basic assessment should be removed (Table 4). A fourth (25.0%) reported that the SCID should be removed and 23.3% the MINI, whereas no one wanted to remove collection of anamnestic information. For the other measures, the two that most participants reported should be removed were the measures that most considered as the least relevant, namely the EQ-5D and CGI-S. Conversely, the two measures that the least number of participants reported should be removed were the measures that the majority considered as the most relevant, namely the AUDIT-C and PHQ-9. A total of 42.1% (*n* = 77) of the participants believed that various measures should be added to the basic assessment.

### The extended diagnostic assessment

A total of 93.0% (*n* = 120) of the participants believed to a large or very large extent that the extended assessment fulfilled its purpose of providing a more elaborate basis for assigning a diagnosis and planning treatment (for means of the extended assessment, see Table 2). There were no significant differences between professions (for comparisons between professions, see Table 5). Of the participants, 70.5% (*n* = 129) had been involved in or conducted the extended diagnostic assessment on their own during the last 12 months. Two thirds (65.9%, *n* = 85) of the 129 participants believed to a large or very large extent that the procedure that the basic assessment could be followed by the extended assessment worked fine. On an interval scale ranging from 1 (*Not at all*) to 5 (*To a very large extent*) participants rated this item *M* = 3.9 (*SD* = 1.0). The mean number of appointments for conducting the extended assessment was 3.7 (*SD* = 2.1) appointments. On average, participants had been involved in 9.5 extended assessments; however, as with the basic assessment, the number of assessments varied greatly across participants and professions (Table 2). There were significant differences in the number of assessments involved in between professions; pairwise comparisons showed a significant difference of a medium to large size between physicians and nurses, indicating that physicians had been involved in more extended assessments than nurses (Table 5). On average, participants had conducted 10.0 extended assessments on their own during the last 12 months; again, the number of assessments varied greatly across participants and professions (Table 2). There were significant differences in the number of assessments conducted on their own between professions; pairwise comparisons showed a significant difference of a medium to large size between physicians and psychologists, indicating that psychologists had conducted more extended assessments on their own than physicians (Table 5).

**Table 5:** Kruskal-Wallis Tests and Pairwise Comparisons of Differences Between Professions of the Extended Assessment

	Kruskal-Wallis test			Physicians-psychologists		Physicians-nurses		Psychologists-nurses	
	*H*	*df*	*p*	*p*	*r*	*p*	*r*	*p*	*r*
Assessment fulfills its purpose	1.25	2	0.534						
Number of assessments involved in	8.41	2	0.015	0.357	0.15	0.019	0.33	0.164	0.25
Number of assessments conducted on their own	17.17	2	< 0.001	< 0.001	-0.37	1.00	0.04	0.060	0.31
I am competent in conducting assessment	7.86	2	0.020	0.016	-0.27	1.00	-0.05	0.919	0.13
I need education in assessment	1.83	2	0.400						
I can determine which diagnosis should be the target of treatment^a^	1.94	2	0.380						
Importance of consulting other clinicians’ assessments	0.37	2	0.832						

^a^“If I have conducted the assessment on my own or substantial parts of it together with others”.

A total of 84.4% (*n* = 108) of the participants considered themselves as competent to a large or very large extent in the extended assessment; however, 35.9% (*n* = 46) believed to a large or very large extent that they needed education in the assessment (Table 2). There were significant differences in perceived competence between professions; pairwise comparisons showed a significant difference of a small to medium size between physicians and psychologists, indicating that psychologists reported higher competence than physicians (Table 5). However, there were no significant differences in the perceived need of education between professions.

If the participants had conducted the extended assessment on their own, or substantial parts of it together with others, and the assessment showed that patients met criteria for multiple diagnoses, 92.2% (*n* = 118) believed to a large or very large extent that they could determine which diagnosis should be the target of treatment (Table 2). There were no significant differences in this perceived ability between professions (Table 5).

A total of 88.6% (*n* = 156) of the participants reported that they considered it important to a large or very large extent to consult other clinicians’ extended assessments prior to starting treatment of patients (Table 2). There were no significant differences between professions (Table 5). Concerning the extended assessment, there were no significant associations of clinicians’ age with their perceived competence, need of education, ability to determine the diagnosis for treatment, or importance of consulting other clinicians’ extended assessments, neither for the entire sample, *r*_*S*_s = -0.02 - -0.11, *p*s = 0.216-0.817, nor separately for physicians, psychologists, and nurses, *r*_*S*_s = -0.03- 0.34, *p*s = 0.054-0.816.

## Discussion

The purpose of the present study was to evaluate clinicians’ perceptions and practices of diagnostic assessment in psychiatric services with the aim of providing a basis for quality improvement. Overall, the results were encouraging in showing that clinicians had positive attitudes toward the basic and extended assessments, and thus SA in general. Furthermore, clinicians considered themselves as competent in the assessments. However, despite local clinical guidelines stating that the clinics are responsible for educating their staff [17], education in the basic assessment seemed rare, and many participants reported that they needed education in the extended assessment.

Although there were some significant differences between physicians and psychologists, differences were most pronounced between nurses and other professions concerning the basic assessment. Nurses had conducted fewer basic assessments on their own than physicians, they perceived themselves as less competent in the basic assessment than psychologists, and they reported to a smaller extent than both physicians and psychologists to be able to determine which diagnosis should be the target for treatment in patients with multiple diagnoses, which is one of the purposes of diagnostic assessment. These differences may be explained by lower actual diagnostic competence in nurses and are expected as diagnostic assessment is not one of the main tasks for this profession. However, although these differences were significant, nurses still perceived themselves as competent to a large extent. Furthermore, nurses believed to a larger extent than physicians that consulting other clinicians’ basic assessments is important prior to starting treatment of patients; however, physicians and psychologists considered this important to a large extent.

In contrast, concerning the extended assessment there were no significant differences between professions in the ability to determine the diagnosis for treatment in patients with multiple diagnoses, or of the importance of consulting other clinicians’ extended assessments prior to starting treatment of patients. An explanation may be that there were only eight nurses that had been involved in the extended assessment, and only eight nurses that had conducted the extended assessment on their own; thus, they may represent a highly selected group. An alternative explanation may be a lack of power to detect significant differences. It should also be noted that physicians had conducted significantly fewer extended assessments on their own than psychologists, and that physicians perceived themselves as less competent than psychologists in the extended assessment. These differences may be explained by the contents of the extended assessment, which may include neuropsychological testing. Because diagnostic assessment often is complex and requires high competence in clinicians [2], psychiatric services in Stockholm might want to consider what qualifications are needed to conduct the basic and extended assessments.

Other studies have also observed differences between professions [1, 11–13], although in different measures than the present study. For example, psychiatrists’ attitudes of the utility of diagnosis have been found to be more positive than in other professions (e.g., counselors, marriage and family therapists), whereas psychologists valued structured diagnostic interviews more than other professions [1]. Furthermore, studies show more frequent use of SA in physicians and psychologists than in other professions (e.g., nurses, social workers), with no difference between physicians and psychologists [11, 12]; however, profession did not moderate the association of attitudes with use [11]. In another study, profession (e.g., psychiatrists, psychologists, nurses) was the only predictor of attitudes toward SA, with counselors being less positive than other professions [13]. To the extent these studies are comparable with each other and the present study, findings are not consistent considering differences in attitudes toward diagnostic and other SA measures between professions, but it seems that physicians and psychologists may hold similar views. However, findings are more consistent in indicating that physicians and psychologists conduct more diagnostic assessment using SA measures than other professions. Thus, previous research and the present study point to the importance of paying attention to potential differences in attitudes and use between professions in efforts to improve quality of assessment and care [23].

Concerning both the basic and extended assessments, there were no significant associations of clinicians’ perceptions with their age, neither for the entire sample nor separately for physicians, psychologists, and nurses. This is consistent with other studies, which equally did not find any associations of age with various perceptions and practices of diagnostic and other SA measures [1, 11–13]. However, it may be more appropriate to associate clinicians’ perceptions with their clinical experience than their age.

Participants assigned varying relevance to the self-report measures and the clinician-administered CGI-S. However, it is not clear why these measures are included in the basic assessment. It can be argued that the measures assessing symptoms of substance use disorder, depression, and ADHD (i.e., AUDIT-C, PHQ-9, and ASRS), respectively, are redundant because these disorders are more thoroughly and objectively assessed by a clinician using a structured diagnostic interview (e.g., MINI or SCID, and, in the case of ADHD, a disorder-specific diagnostic interview as part of the extended assessment). Moreover, these measures are primarily used as screening tools and to assess symptoms, not to make a diagnosis [24–26]. Similarly, the measures assessing health-related quality of life (i.e., EQ-5D) and illness severity (i.e., CGI-S) do not provide a basis for assigning a diagnosis because quality of life or illness severity do not constitute specific diagnostic criteria. Perhaps the reason to why these measures are included in the basic assessment can be found in the local clinical guidelines [17], which state that the basic assessment should “enable systematic and comparable evaluation of care and treatment results” (p. 3). If so, this is problematic for two reasons: (a) the measures are not administered in direct connection to treatment start, which makes impossible a correct assessment of current symptomatic status, and (b) diagnosis-specific symptoms measures for most psychiatric disorders are lacking. Moreover, the basic assessment does not include a measure of functional impairment, arguably a relevant outcome measure [27]. Thus, the self-report measures and the CGI-S may not be necessary in the basic assessment because, in most cases, anamnestic information and information collected using the MINI or SCID should provide a sufficient basis for assigning a diagnosis. Therefore, psychiatric services in Stockholm might want to consider whether to exclude these measures from the basic assessment.

However, self-report and clinician-administered measures should be included in mandatory evaluation of treatment. Evaluation should include administration of outcome measures before and regularly during treatment to inform treatment decisions, consistent with measurement-based care [28]. Unfortunately, despite support for improving patient care, including progress monitoring, symptom reduction, and detection of deterioration, measurement-based care is underutilized in routine clinical practice [29]. Evaluation should also include administration of outcome measures before and following treatment to evaluate treatment effects for assessing and improving the quality of care [30]. As with measurement-based care, evaluation of treatment effects is infrequent in psychiatric services [31].

### Limitations

The present study had some limitations which should be considered when interpreting the results. First, with a response rate of 53.2% only half of the eligible clinicians participated in the survey. This may indicate that those who participated were clinicians with a selected set of specific perceptions or practices. Second, eligible participants were clinicians who had conducted assessment during the last 12 months and documented it in the medical record system, which means that clinicians who had conducted assessment earlier or not documented it were excluded. However, it was considered important that the participants had an up-to-date view of diagnostic assessment. Moreover, reportedly, it is uncommon that clinicians conduct assessment without documenting it in the medical record system. Third, the survey was developed for the present study and its psychometric properties have not been investigated. Finally, it is important to recognize that the present study was based on self-reported data and that it did not examine, for example, actual diagnostic competence or need of education, or issues of reliability (e.g., whether clinicians are conducting assessment in a similar manner) or validity (e.g., whether the basic and extended assessments are better or worse than alternative procedures of diagnostic assessment).

## Conclusions

The present study showed that clinicians in a community-based psychiatric service in general had positive attitudes toward diagnostic assessment. However, some potential areas of improvement were identified, including clinician qualifications, education in diagnostic assessment, and contents of diagnostic assessment. The results of the present study may provide a basis for quality improvement in psychiatric services.

## Data Availability

The dataset used and analyzed during the present study is available from the author (BB) on reasonable request, provided that the clinics approve of disclosure.

## List of abbreviations

ADHD: Attention-deficit/hyperactivity disorder.
ASRS: Adult ADHD Self-Report Scale.
AUDIT-C: Alcohol Use Disorders Identification Test-Consumption.
CGI-S: Clinical Global Impression-Severity.
EQ-5D: EuroQol Five Dimensional Questionnaire.
MINI: Mini International Neuropsychiatric Interview.
PHQ-9: Patient Health Questionnaire-9.
SA: Standardized assessment.
SCID: Structured Clinical Interview for Diagnostic and Statistical Manual of Mental Disorders.
